# Effect of Insulin Analogues on Insulin/IGF1 Hybrid Receptors: Increased Activation by Glargine but Not by Its Metabolites M1 and M2

**DOI:** 10.1371/journal.pone.0041992

**Published:** 2012-07-26

**Authors:** Cécile Pierre-Eugene, Patrick Pagesy, Tuyet Thu Nguyen, Marion Neuillé, Georg Tschank, Norbert Tennagels, Cornelia Hampe, Tarik Issad

**Affiliations:** 1 Institut Cochin, Université Paris Descartes, CNRS (UMR8104), Paris, France; 2 INSERM, U1016, Paris, France; 3 Sanofi-Aventis, Frankfurt, Germany; University of Tor Vergata, Italy

## Abstract

**Background:**

In diabetic patients, the pharmacokinetics of injected human insulin does not permit optimal control of glycemia. Fast and slow acting insulin analogues have been developed, but they may have adverse properties, such as increased mitogenic or anti-apoptotic signaling. Insulin/IGF1 hybrid receptors (IR/IGF1R), present in most tissues, have been proposed to transmit biological effects close to those of IGF1R. However, the study of hybrid receptors is difficult because of the presence of IR and IGF1R homodimers. Our objective was to perform the first study on the pharmacological properties of the five marketed insulin analogues towards IR/IGF1R hybrids.

**Methodology:**

To study the effect of insulin analogues on IR/IGF1R hybrids, we used our previously developed Bioluminescence Resonance Energy Transfer (BRET) assay that permits specific analysis of the pharmacological properties of hybrid receptors. Moreover, we have developed a new, highly sensitive BRET-based assay to monitor phophatidylinositol-3 phosphate (PIP_3_) production in living cells. Using this assay, we performed a detailed pharmacological analysis of PIP_3_ production induced by IGF1, insulin and insulin analogues in living breast cancer-derived MCF-7 and MDA-MB231 cells.

**Results:**

Among the five insulin analogues tested, only glargine stimulated IR/IGF1R hybrids with an EC50 that was significantly lower than insulin and close to that of IGF1. Glargine more efficiently stimulated PIP_3_ production in MCF-7 cells but not in MDA-MB231 cells as compared to insulin. In contrast, glargine metabolites M1 and M2 showed lower potency for hybrid receptors stimulation, PIP_3_ production, Akt and Erk1/2 phosphorylation and DNA synthesis in MCF-7 cells, compared to insulin.

**Conclusion:**

Glargine, possibly acting through IR/IGF1R hybrids, displays higher potency, whereas its metabolites M1 and M2 display lower potency than insulin for the stimulation of proliferative/anti-apoptotic pathways in MCF-7 cells.

## Introduction

The link between the quality of glycemic control and diabetic complications is now clearly established. However, in diabetic patients, injections of human insulin do not achieve an optimal control of glycemia. Indeed, in normal individuals, food intake induces a rapid increase in plasma insulin, which reaches its maximal level after 30 to 45 min. Insulin concentration then decreases to reach its basal level within 2 to 3 h. However, in diabetic patients, injection of human insulin does not mimic this profile [1]. Indeed, insulin has a tendency to self-associate and it is found in a hexameric form when injected in the subcutaneous tissues. Insulin can only be absorbed through the capillary wall into the circulation in a monomeric form, therefore the appearance of injected insulin in the blood is delayed [1], [2]. This can result in post-prandial hyperglycaemia as well as increased risk of hypoglycemia before the following meal. Insulin analogues, displaying either slow (glargine, detemir) or rapid pharmacokinetics (aspart, lispro, glulisine) have been developed to mimic basal insulin levels and rapid insulin secretion peaks that occur after eating. However, concerns have been raised about the potential mitogenic and anti-apoptotic properties of fast- and slow-acting analogues [3]–[5]. Moreover, recent epidemiological studies gave contradictory results concerning a potential association between the use of glargine and cancer risk, notably breast cancer [6]–[8]. The controversy raised by these studies underlines the importance of a detailed characterization of the pharmacological properties of these insulin analogues.

The properties of these analogues towards insulin receptors (IR) and IGF1 receptors (IGF1R) have already been evaluated [3], [9], however their potential effects on insulin/IGF1 hybrid receptors still remains a major question. The IR and the IGF1R are both heterotetramers consisting of one αß subunit pair complexed with another αß subunit pair ((αß)_2_). The α-subunits are extracellular and bind ligands, whereas the ß-subunits possess an intracellular tyrosine-kinase activity [10]. A large number of studies have demonstrated the existence of IR/IGF1R hybrid receptors, consisting of an αß subunit pair of the IR associated with an αß subunit pair of the IGF1R [11], [12], in both normal [13], [14] and pathological situations, including diabetes [15] and cancer [16]. In tissues from insulin resistant or diabetic patients, the expression of IR/IGF1R hybrids is increased while IR expression is decreased [15], [17]–[19]. This may contribute to decreased insulin sensitivity in these patients, since hybrid receptors were shown to display lower affinity for insulin and higher affinity for IGF1 [18], [20]. Due to these “IGF1R-like” properties, hybrid receptors may also play a role in breast cancer [16], thyroid cancer [21] and colonic cancer cells [22]. In view of their “IGF1R-like” properties and increased expression in diabetic patients, it is of considerable importance to evaluate the pharmacology of insulin analogues on IR/IGF1R hybrids. However, these hybrids are technically difficult to study because cells expressing hybrid receptors also express homodimeric IR and IGF1R.

**Figure 1:**
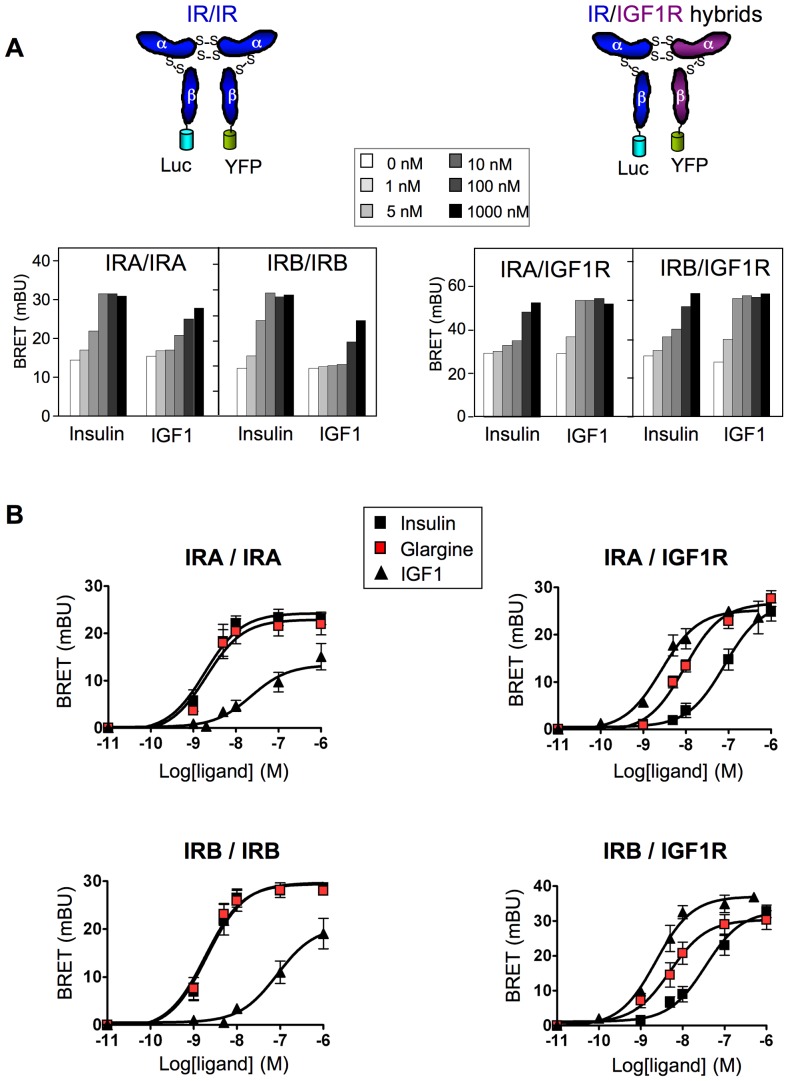
Effect of insulin, glargine and IGF1 on IR homodimers and IR/IGF1R hybrid receptors. HEK-293 cells were co-transfected with IRA-Luc/IRA-YFP, IRB-Luc/IRB-YFP, IRA-Luc/IGF1R-YFP or IRB-Luc/IGF1R-YFP. Receptors were partially purified by WGL chromatography. Ligand binding induces a conformational change that brings the two ß-subunits in close proximity, resulting in an energy transfer between the luciferase and YFP. BRET assays were performed in the presence of increasing ligand concentrations. (A) Typical experiments showing basal and insulin or IGF1 stimulated BRET signals. (B) Dose-response curves showing the effect of insulin, IGF1 and glargine on IR homodimers and IR/IGF1R hybrids. Ligand-induced BRET (BRET above basal) was determined at each ligand concentration and was used to establish dose-response curves. Results are the means ± S.E.M. of 4 to 6 independent experiments. EC50 for insulin, IGF1, glargine and other insulin analogues are given in Table 1.

We previously developed a BRET-based method to monitor ligand-induced conformational changes within the IR [23], [24]. By generating insulin receptors with one ß-subunit fused to *Renilla* luciferase (Luc) and the other ß-subunit fused to yellow fluorescent protein (YFP) (Fig. 1A), we demonstrated that ligand-induced conformational changes produced a BRET signal that reflects the activation state of the receptor [23]. More recently, we demonstrated that this method constitutes a unique tool to specifically monitor the activation of IR/IGF1R hybrids [25], [26] by utilizing constructs where the ß-subunit of the IR is fused to Luc and the ß-subunit of the IGF1R is fused to YFP (Fig. 1A). With this method only hybrid receptors are BRET competent, which allows specific pharmacological studies on these hybrids [25]. In the present study, we took advantage of this method to establish, for the first time, the pharmacological profile of insulin analogues towards insulin/IGF1 hybrid receptors. Increased risk of breast cancer has been associated with diabetes in numerous epidemiological studies [27], therefore we also studied the effect of insulin analogues on the production of PIP_3_ in breast cancer derived cell lines using a new, highly sensitive BRET-based assay.

## Methods

### Reagents

All chemical reagents have been described previously [23], [25], [28]. Human insulin (actrapid) and human insulin analogues [glargine (A21Gly,B31Arg,B32Arg-insulin), detemir (B29Lys(e-tetradecanoyl),desB30)-insulin), lispro (B28Lys,B29Pro-insulin), glulisine (B3Lys,B29Glu-insulin) and aspart (B28Asp-insulin)] were kind gifts from Profs. B. Fève, J. Bertherat and J-D. Chiche (AP–HP, Paris). Glargine metabolites, M1 (A21Gly-insulin) and M2 (A21Gly,B30desThr-insulin) were made available by Process Development Biotechnology (Sanofi-Aventis, Frankfurt, Germany). Immunoprecipitation of IR and IR/IGF1R hybrids was performed using a monoclonal anti-IR antibody (CT1) covalently bound to sepharose beads [29]. Immunoblotting was performed using anti-IR (Santa Cruz C-19), anti-IGF1R (Santa Cruz C-20), anti-Erk2 (Santa-Cruz C-14), anti-Akt (Santa-Cruz H-136), anti-phospho-Erk (Cell Signaling 9101) and anti-phospho-Akt (Cell Signaling 9271) antibodies.

### Expression vectors

cDNAs coding for Luc- or YFP-tagged receptors have been described previously [23], [25], [30]. The cDNA coding for YFP-targeted to the plasma membrane (pEYFP-Mem) was from Clontech. The cDNA coding for Luc-Akt-PH was obtained by fusing the Pleckstrin Homology (PH) domain of mouse Akt1 to the C-terminus of *Renilla* luciferase.

### Cell culture, transfection, partial purification of receptors and BRET assays

Culture of HEK-293 cells, transfection and purification of receptors by wheat-germ lectin (WGL) chromatography have been described previously [23]. MCF-7 and MDA-MB-231 cells were cultured as described previously [31], [32]. For studies of PIP_3_ production, MCF-7 and MDA-MB-231 cells were transfected with 0.7 mg Luc-Akt-PH and 0.3 mg pYFP-Mem cDNAs per 10.3 mm dish and transferred in a 96 well plate 24 h before BRET experiments. BRET experiments were performed exactly as described previously [23], [28], [33].

### Akt and Erk1/2 phosphorylation, gene expression and thymidine incorporation in MCF-7 cells

Akt and Erk1/2 phosphorylation were evaluated both by classical western-blotting [34] and by using in-cell western as described previously [35]. Total RNA for quantitative PCR was isolated [36] and reverse-transcribed as described previously [37]. Quantitative PCR was performed using a Lightcycler system and SYBR Green I using the following primer sequences: EGR1: Forward: GCACCTGACCGCAGAGTCTT, Reverse: AGTGGTTTGGCTGGGGTAACT; IGFBP1 Forward: TATGATGGCTCGAAGGCTCT, Reverse, TAGACGCACCAGCAGAGTCC; Cyclophyline A Forward: GGTGACTTCA CACGCCATAATG, reverse, ACAAGATGCCAGGACCCGTAT. DNA synthesis was determined by [^14^C]thymidine incorporation in MCF-7 cells as described previously [38].

### Statistical analysis

Determination of EC50 was performed with Prism software by non-linear regression analysis of the dose-response curves using a 4 parameter logistics model. Statistical analysis was performed using ANOVA followed by Dunnett's post-test.

## Results

### Pharmacological properties of insulin analogues towards the IR/IGF1R hybrids

Alternative splicing of the IR mRNA results in two isoforms which differ by the absence (IRA) or presence (IRB) of 12 amino acids located at the C-terminal end of the α-subunit. Since these isoforms display different biological and pharmacological properties [39], [40], we studied the effect of insulin analogues towards IRA and IRB homodimers as well as IRA/IGF1R and IRB/IGF1R hybrids. As described previously, insulin stimulated the IR homodimers more efficiently than IGF1 [23], whereas IGF1 was more efficient on IR/IGF1R hybrids [25] (Fig. 1A).

We then evaluated the effect of the five marketed insulin analogues towards IR homodimers and IR/IGF1R hybrids (Fig. 1B and Table 1). Interestingly, whereas glargine's pharmacological profile towards IR homodimers was superimposable to that of insulin, its potency towards both types of hybrid receptors was significantly higher compared to insulin (Table 1). No significant differences in pharmacological profiles were observed for the other insulin analogues, although lispro tended to have a slightly higher potency than insulin towards IR/IGF1R hybrids, and detemir tended to have a lower potency on homodimers and hybrid receptors (Table 1). Thus, among the five analogues used for the treatment of diabetes, only glargine showed a profile similar to that of IGF1 towards hybrid receptors.

**Table 1:** EC50s of insulin, insulin analogues and IGF1 towards IR homodimers and IR/IGF1R hybrids.

	IRA/IRA	IRB/IRB	IRA/IGF1R	IRB/IGF1R
	*EC50 (nM)*	*EC50 (nM)*	*EC50 (nM)*	*EC50 (nM)*
**Insulin**	2.70±0.65	2.56±0.69	130.28±41.05	69.61±34.60
**IGF1**	34.38±13.36 **	50.03±12.89 **	3.01±0.66 **	2.87±0.66 **
**Glargine**	2.38±0.32	2.39±0.50	18.07±8.54 **	7.60±2.99 **
**Detemir**	5.13±2.75	5.24±1.79	335.39±224.19	174.99±42.61
**Aspart**	1.48±0.26	2.97±1.50	127.62±36.23	34.13±10.51
**Glulisine**	1.61±0.39	1.64±0.33	183.22±63.08	107.48±41.15
**Lispro**	1.65±0.10	2.46±0.94	55.67±22.78	22.97±14.62

Results are the means ± S.E.M. of 4 to 6 independent experiments.

** : statistically significant differences at p<0.01 when compared to insulin.

### Effect of insulin analogues on PIP_3_ production in living cells

**Figure 2:**
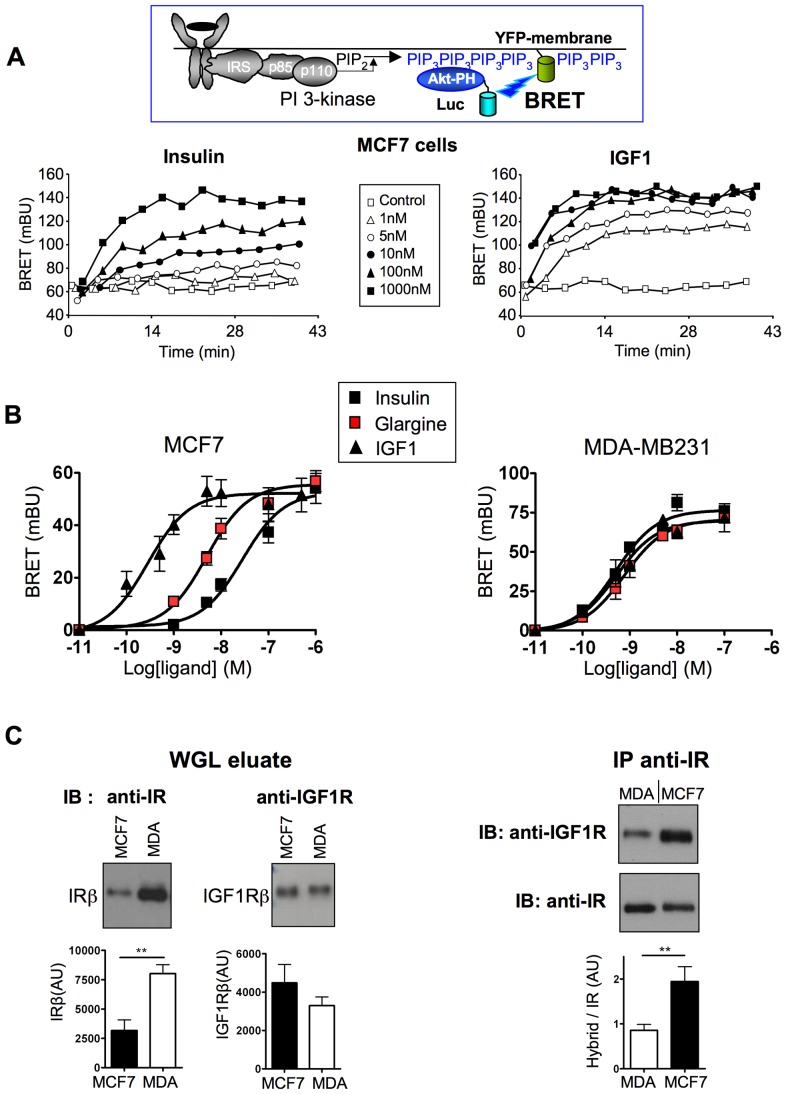
Effect of insulin, glargine and IGF1 on PIP_3_ production in intact living cells. Activation of tyrosine kinase receptors by their ligands stimulates the activity of PI-3 kinase, leading to increased phosphorylation of phosphatidyl-inositol 2 phosphate (PIP_2_) into phosphatidyl-inositol 3 phosphate (PIP_3_) and subsequent recruitment of Akt to the plasma membrane through its pleckstrin homology (PH) domain. To monitor the production of PIP_3_ induced by receptor activation, cells were co-transfected with cDNAs coding for the PH domain of Akt fused to luciferase (Luc-Akt-PH) and YFP fused to the membrane localization sequence of neuromodulin. Cells were pre-incubated for 10 min with coelenterazine and then stimulated with increasing ligand concentrations. (A) Typical experiment showing real-time insulin or IGF1 effects on PIP_3_ production in MCF-7 cells. (B) Dose-dependent effect of insulin, glargine and IGF1 on PIP_3_ production in MCF-7 and MDA-MB231 cells. Ligand-induced BRET (BRET above basal at the plateau) was determined for each ligand concentration and was used to establish dose-response curves. Results are the means ± S.E.M. of 5 to 6 independent experiments. EC50 for insulin, IGF1, glargine and other insulin analogues are given in Table 2. (C) Left panel: Receptors were partially purified from MDA-MB231 and MCF-7 cells by WGL chromatography. WGL eluates (12 µg of protein) were submitted to electrophoresis and western-blotting. IR and IGF1R expression levels were evaluated by immunoblotting (IB) using anti-IR (C-19) and anti-IGF1R (C-20) antibodies. Right panel: After normalization of the eluates for IR content, immunoprecipitation (IP) was performed using anti-IR antibody (CT1) and hybrid receptors were detected using anti-IGF1R antibody. Blots were then stripped and reprobed with anti-IR antibody. Results are representative of 6 immunoprecipitation experiments performed on three independent batches of receptor preparations (**, p<0.01).

We then studied the effects of these ligands on PIP_3_ production induced by endogenous receptors in living cells, using a new BRET assay (Fig. 2A). This assay is based on the recruitment of the PH domain of Akt (Akt-PH) to the plasma membrane in response to PI-3 kinase-induced PIP_3_ production. We observed that insulin and IGF-1 stimulated BRET in a dose dependent manner (Fig. 2A). This effect was inhibited by the PI-3 kinase inhibitor LY294002 (Fig. S1) indicating that the BRET signal measured in these experiments indeed reflected PIP_3_ generated by activation of PI-3 kinase.

Using this method, we studied the pharmacological profile of IGF1, insulin and insulin analogues on PIP_3_ production in MCF-7 and MDA-MB231 breast cancer cells (Fig. 2B and Table 2). In MCF-7 cells, IGF-1 was much more potent than insulin for activation of PIP_3_ production. Among the five insulin analogues, only glargine stimulated PIP_3_ production with higher potency compared to insulin (Fig. 2B and Table 2). In contrast, in MDA-MB231 cells, insulin and insulin analogues stimulated PIP_3_ production with similar high-affinity (Fig. 2B and Table 2), suggesting that their effects were mediated by IR in these cells.

**Table 2:** EC50s of insulin, insulin analogues and IGF1 for PIP_3_ production in MCF-7 and MDA-MB231 cells.

	MCF-7	MDA-MB231
	*EC50 (nM)*	*EC50 (nM)*
**Insulin**	38.21±8.32	0.55±0.07
**IGF1**	0.44±0.08 **	0.71±0.10
**Glargine**	4.64±0.75 **	0.78±0.18
**Detemir**	164.68±104.42	2.30±0.18 **
**Aspart**	43.41±27.64	0.59±0.10
**Glulisine**	124.23±25.20 *	0.84±0.18
**Lispro**	58.48±25.90	0.43±0.13

Results are the means ± S.E.M. of 5 to 6 independent experiments.

*, ** , p<0.05 or p<0.01 respectively, when compared to insulin.

To determine whether the different results obtained in the two cell lines were due to differences in IGF1R, IR or hybrid receptor expression, receptors were partially purified from MCF-7 and MDA-MB231 cells by WGL chromatography. Western-blotting experiments indicated that the expression of IGF1R was similar in both cell lines, whereas the expression of IR was significantly higher in MDA-MB231 cells (Fig. 2C, left panel). To detect hybrid receptors, IR were immunoprecipitated from WGL eluates using an anti-IR antibody and submitted to western blotting using an anti-IGF1R antibody. The specificity of this procedure for the detection of hybrid receptors was verified in previous experiments using IR-Luc/IGF1R-YFP hybrids (Fig. S2). Then, equivalent amounts of IR from MCF-7 or MDA-MB231 cells were immunoprecipitated using the anti-IR antibody and immunodetected with the anti-IGF1R antibody (Fig. 2C, right panel). We observed that the amount of IGF1R precipitated with anti-IR antibody (i.e., the relative amount of IR engaged in IR/IGF1R hybrids) was two-fold higher in MCF-7 than in MDA-MB231 cells, in agreement with previous results [16].

### Effect of glargine metabolites M1 and M2 on hybrid receptors and PIP_3_ production in MCF-7 cells

**Figure 3:**
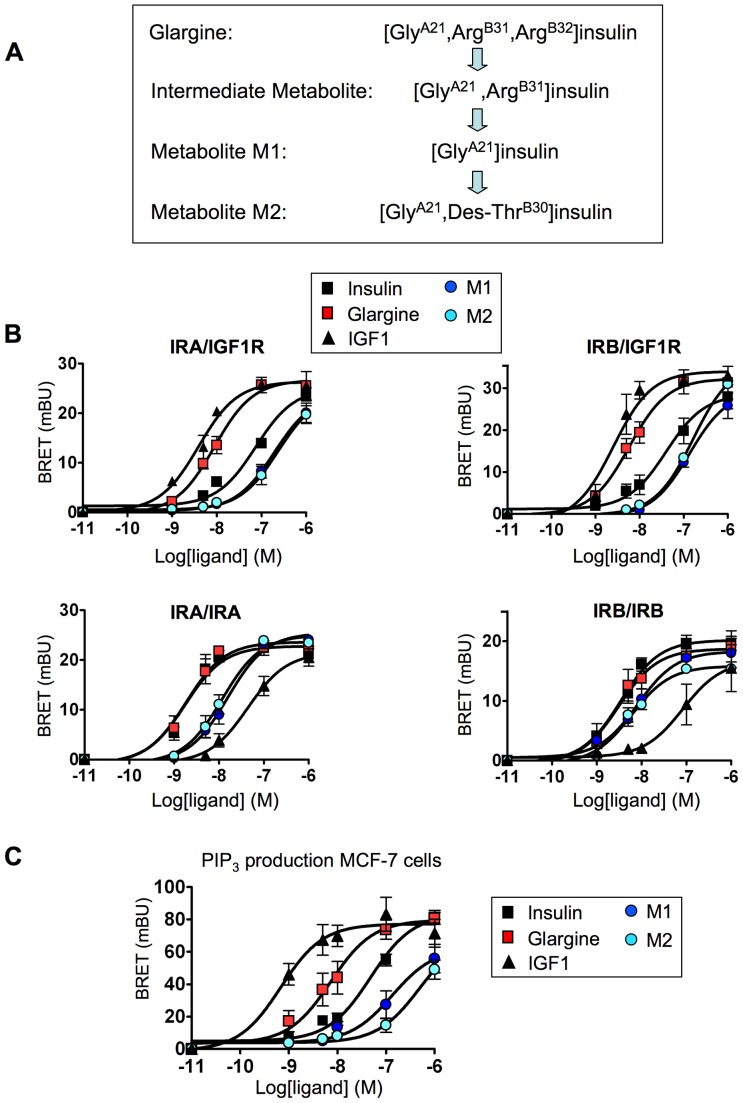
Comparison of the pharmacological profiles of glargine and its metabolites M1 and M2. (A) Conversion of glargine into M1 and M2 metabolites. (B) Effects of M1 and M2 on IR/IGF1R hybrids and on IR/IR homodimers. Receptors were prepared as described in figure 1. BRET assays were performed in the presence of increasing concentrations of insulin, glargine, M1, M2 or IGF1. (C) Dose-dependent effect of insulin, glargine, M1, M2 or IGF1 on PIP_3_ production in MCF-7 cells. BRET assays were performed in the presence of increasing concentrations of ligands. Ligand-induced BRET (BRET above basal at the plateau) was determined for each ligand concentration and was used to establish dose-response curves. Results are the means ± S.E.M. of 4 to 6 independent experiments. EC50 for insulin, IGF1, glargine and its metabolites are given in Table 3 and 4.

Previous studies indicated that *in vivo*, glargine is converted into metabolites M1 and M2 (Figure 3A) [41], [42], which have a metabolic potency comparable to that of insulin but a lower growth-promoting activity than insulin [38]. Therefore, we evaluated the effect of M1 and M2 on hybrid receptors. We observed that, in contrast to glargine, M1 and M2 were less efficient than insulin in stimulating IRA/IGF1R and IRB/IGF1R hybrids (Fig. 3B and Table 3). M1 and M2 were also less efficient than insulin in stimulating IRA homodimers, suggesting a decreased affinity towards this isoform. We also evaluated the effect of M1 and M2 on PIP_3_ production in MCF-7 cells, previously shown to be more sensitive to glargine than to insulin. We observed a lower potency of M1 and M2 metabolites in stimulating PIP_3_ production in MCF-7 cells compared to insulin and glargine (Fig. 3C and Table 4).

**Table 3:** EC50s of insulin, IGF1, glargine and glargine metabolites towards IR homodimers and IR/IGF1R hybrids.

	IRA/IRA	IRB/IRB	IRA/IGF1R	IRB/IGF1R
	*EC50 (nM)*	*EC50 (nM)*	*EC50 (nM)*	*EC50 (nM)*
**Insulin**	2.15±0.40	3.68±1.13	84.30±22.74	48.15±13.72
**Glargine**	2.04±0.50	3,88±1.25	10.19±1.29 **	6.28±1.37 **
**M1**	16.69±3.16 **	9.64±2.18	225.40±47.69	147.08±35.25
**M2**	13.81±2.47 **	6.31±0.98	314.60±71.85 *	169.20±18.87 *
**IGF1**	50.63±13.45 **	130.04±60,95 **	4.23±0.82 **	3.05±1.10 **

Results are the means ± S.E.M. of 4 to 6 independent experiments.

*, ** , p<0.05 or p<0.01 respectively, when compared to insulin.

**Table 4:** EC50s of insulin, IGF1, glargine and glargine metabolites for PIP_3_ production, Akt and Erk activation and thymidine incorporation in MCF-7 cells.

	pAkt	pERK1/2	PIP_3_ MCF-7	[^14^C]thymidine MCF-7
	*EC50 (nM)*	*EC50 (nM)*	*EC50 (nM)*	*EC50 (nM)*
**Insulin**	35.6±7.7	107±28	44.91±8.93	15.94±3.51
**Glargine**	4.37±1.09 **	24.9±6.6 *	9.33±3.18 *	3.89±0.64 **
**M1**	42.52±9.29	211±53	307.88±84.36 *	29.30±1.53*
**M2**	44.54±10.52	170±50	341.12±122.26 *	20.00±1.25
**IGF1**	0.67±0.26 **	6.7±2.2 **	0.76±0.23 **	0.42±0.04 **

Results are the means ± S.E.M. of 4 to 6 independent experiments.

*, ** , p<0.05 or p<0.01 respectively, when compared to insulin.

### Effect of glargine and its metabolites M1 and M2 on downstream signaling events in MCF-7 cells

**Figure 4:**
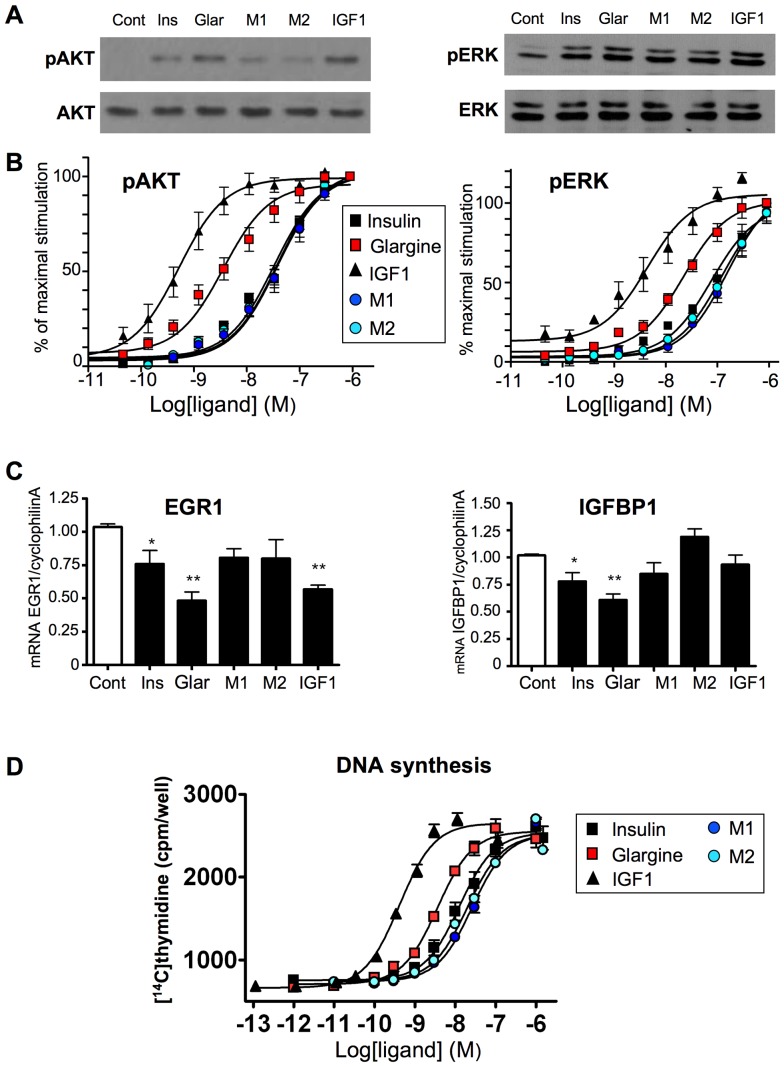
Downstream biological effects of insulin, glargine and its metabolites M1 and M2 in MCF-7 cells. (A) Effect of insulin, IGF1, glargine and its metabolites M1 and M2 on Akt and Erk1/2 phosphorylation in MCF-7 cells. MCF-7 cells were starved overnight and then incubated for 5 min in presence of 10 nM of insulin, glargine, M1, M2 or IGF1. Ligand-induced phosphorylation of Erk1/2 and Akt was evaluated by western blotting. (B) Dose-dependent effect of insulin, IGF1, glargine and its metabolites M1 and M2 on Akt and Erk1/2 phosphorylation in MCF-7 cells. Cells were stimulated for 20 min and ligand-induced phosphorylation of Akt and Erk1/2 was evaluated by in-cell western. Results correspond to mean ± SEM of 4 to 6 independent experiments. (C) MCF-7 cells were incubated for 18 h in serum free medium in the presence or absence of 10 nM of insulin, glargine, M1, M2 or IGF1. mRNA expression level was measured by qRTPCR. Results are normalized to the expression of cyclophilin A mRNA and correspond to the mean ± SEM of 4 to 8 independent experiments (*, **, p<0.05 or p<0.01 respectively, when compared to the control condition). (D) Subconfluent MCF-7 cells cultured in Cytostar-T scintillation microplates were starved for 4 h and then incubated for 19 h with increasing concentrations of IGF-1, insulin or analogues in serum free medium. [^14^C]thymidine was added for an additional 6 h and the radioactivity measured in a Wallac 1450 Micro Beta Trilux Scintillation counter. Data are means ± SEM of at least 6 independent experiments. EC50 for insulin, IGF1, glargine and its metabolites on Akt and Erk1/2 phosphorylation and on thymidine incroporation are given in Table 4.

We compared the effects of insulin, glargine, M1, M2 and IGF1 on Akt and Erk1/2 phosphorylation in MCF-7 cells using both western-blot (Fig. 4A) and in-cell western (Fig. 4B). In agreement with the results obtained with these ligands in BRET experiments (Fig. 3), glargine stimulated Akt and Erk with significantly higher potency compared to insulin, whereas the effects of M1 and M2 were similar to those of insulin (Fig. 4A, B and Table 4). Using quantitative RT-PCR, we also evaluated the effect of these ligands on the expression of two genes involved in the regulation of cell proliferation (Fig. 4C). EGR1 is a transcription factor that acts as a tumor suppressor in breast cancer cells [43], whereas IGFBP1 regulates cell proliferation by binding to and inhibiting IGF1 effects [44]. We observed that the expression of EGR1 and IGFBP1 was significantly inhibited by overnight treatment with 10 nM glargine. Inhibition by insulin was less marked, whereas M1 and M2 had no significant effect. In agreement with these results, glargine stimulated thymidine incorporation into DNA with higher potency, whereas M1 and M2 displayed similar or lower potency than insulin (Fig. 4D and Table 4).

## Discussion

Insulin analogues are widely used for the treatment of millions of diabetic patients, but their effects on IR/IGF1R hybrids had never been studied. Expression of hybrid receptors have been detected in human skeletal muscle, heart, coronary artery smooth muscle cells, endothelial cells, adipose tissue, fibroblasts, spleen, red and white blood cells and placenta [13], [14], [45], [46]. Since the expression of IR/IGF1R hybrids appears to be increased in tissues from diabetic patients [15], [18], [19], it is highly important to determine the pharmacological properties of marketed insulin analogues towards these hybrids. Indeed, in addition to potential pro-mitogenic effects associated with IR/IGF1R stimulation, undesirable effects in some tissues may also occur even in fully differentiated, non-proliferating cells. For instance, in 3T3L1 adipocytes, the proportion of IR/IGF1R increases during differentiation, and activation of these receptors in mature adipocytes stimulates glucose uptake [47]. Thus, differential pharmacodynamic or pharmacokinetic activities of insulin analogues in diabetic patients, which over-express hybrid receptors in adipose tissue [19], may influence weight gain associated with insulin therapy [48].

In the present study, to establish the pharmacological profile of these analogues, we have used a unique BRET-based assay that specifically monitors the effect of different ligands on the activity of IR/IGF1R hybrids. We show that among the five insulin analogues presently used to treat diabetes, only glargine displays a significantly higher potency than insulin in stimulating IRA/IGF1R and IRB/IGF1R (Fig. 1B and Table 1). Interestingly, we observed a tendency towards an increased potency of lispro on IRB/IGFR, which might deserve further investigation as increased proliferation rates have been reported for this ligand in different cell lines [4], [49].

We also introduced a new, highly sensitive BRET assay to monitor PIP_3_ production induced by activation of endogenous receptors in living cells. The sensitivity and robustness of this assay permit the establishment of the pharmacological profile of activation of this pathway by different ligands. In contrast to MDA-MB231 cells, we observed that glargine stimulated PIP_3_ production with higher potency than insulin in MCF-7 cells. Moreover, in these cells, downstream effects of glargine, including phosphorylation of Akt and Erk, inhibition of anti-proliferative gene expression and stimulation of DNA synthesis were more pronounced. These effects could be mediated by IR/IGF1R hybrids, which are more sensitive to glargine than to insulin (Fig. 1B). Indeed, we observed that in MCF-7 cells, relatively high amounts of IR are engaged in IR/IGF1R hybrids compared to MDA-MB231 cells (Fig. 2C). However, other mechanisms, including subtle differences in IGF1R, IRA or IRB expression levels or in signal transduction efficiency in the two cell lines may also play a role in the differential effects of glargine.

Although glargine's potency towards hybrid receptors is higher than that of insulin, it is important to note that its EC50 towards hybrids is far above peak serum levels (about 200–300 pM) reached after injection in diabetic patients [50]–[52]. *In vivo*, glargine was shown to be converted into active metabolites M1 and M2 [41], [42]. We observed a lower potency of M1 and M2 towards IR/IGF1R hybrids (Fig. 3B) compared to insulin and glargine. Similar results were obtained in MCF-7 cells for the stimulation of PIP_3_ production (Fig. 3C), Akt and Erk phosphorylation, gene expression and DNA synthesis (Fig. 4). Therefore, whereas glargine may show pro-mitogenic properties in cultured cells, these properties should be abrogated *in vivo* if glargine is rapidly converted into M1 and M2, as suggested by previous studies [41], [42].

## Supporting Information

Figure S1 **Inhibition of insulin and IGF1-induced BRET by the PI-3 kinase inhibitor LY294002.** HEK-293 cells co-transfected with Luc-Akt-PH and Mem-EYFP were pre-incubated for 1 h in presence of 50 mM LY294002 or vehicle (DMSO). After addition of coelenterazine, cells were stimulated with 100 nM insulin or IGF1 and BRET measurements were performed in real time during more than 30 min. Basal and ligand-induced BRET were markedly inhibited by LY294002, indicating that these signals reflect the activity of PI-3 kinase in the cell.
(TIF)

Figure S2 **Validation of the method used for detection of IR/IGF1R hybrids.** Partially purified receptors prepared from HEK-293 cells transfected as described in Fig. 1 were used to establish the specificity of the immunoprecipitation and immunoblotting experiments. (A) WGL eluates were submitted to SDS-PAGE followed by immunoblotting (IB) using anti-IR (Santa Cruz C19) or anti-IGF1R antibodies (Santa Cruz C-20). (B) Luciferase and YFP-tagged IR, IGF1R and hybrid receptors were immunoprecipitated (IP) using an anti-IR antibody (CT1) immobilized on sepharose beads, submitted to SDS-PAGE followed by immunoblotting using anti-IGF1R (Santa Cruz C-20) and anti-IR (Santa Cruz C-19) antibodies. After immunoprecipitation with anti-IR antibody, only hybrid receptors were detected when immunoblotting with anti-IGF1R antibody.
(TIF)
