# Supplementary figures and images for: Effect of Insulin Analogues on Insulin/IGF1 Hybrid Receptors: Increased Activation by Glargine but Not by Its Metabolites M1 and M2
Source: PLoS One. 2012 Jul 26;7(7):e41992. doi: 10.1371/journal.pone.0041992 (PMC3406060; doi:10.1371/journal.pone.0041992)

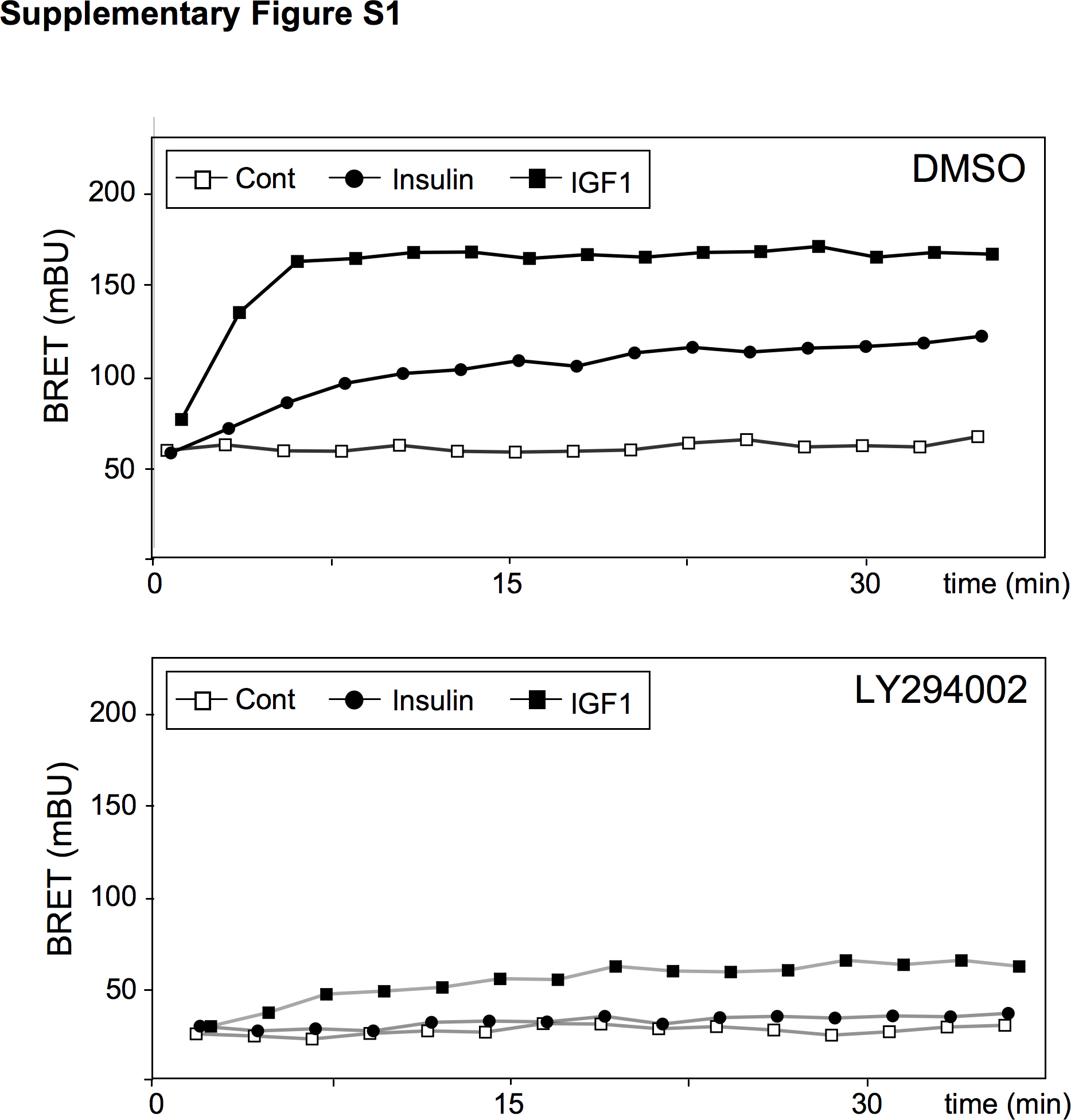

Supplement: Figure S1 — Inhibition of insulin and IGF1-induced BRET by the PI-3 kinase inhibitor LY294002. HEK-293 cells co-transfected with Luc-Akt-PH and Mem-EYFP were pre-incubated for 1 h in presence of 50 mM LY294002 or vehicle (DMSO). After addition of coelenterazine, cells were stimulated with 100 nM insulin or IGF1 and BRET measurements were performed in real time during more than 30 min. Basal and ligand-induced BRET were markedly inhibited by LY294002, indicating that these signals reflect the activity of PI-3 kinase in the cell. (TIF) [file pone.0041992.s001.tif]

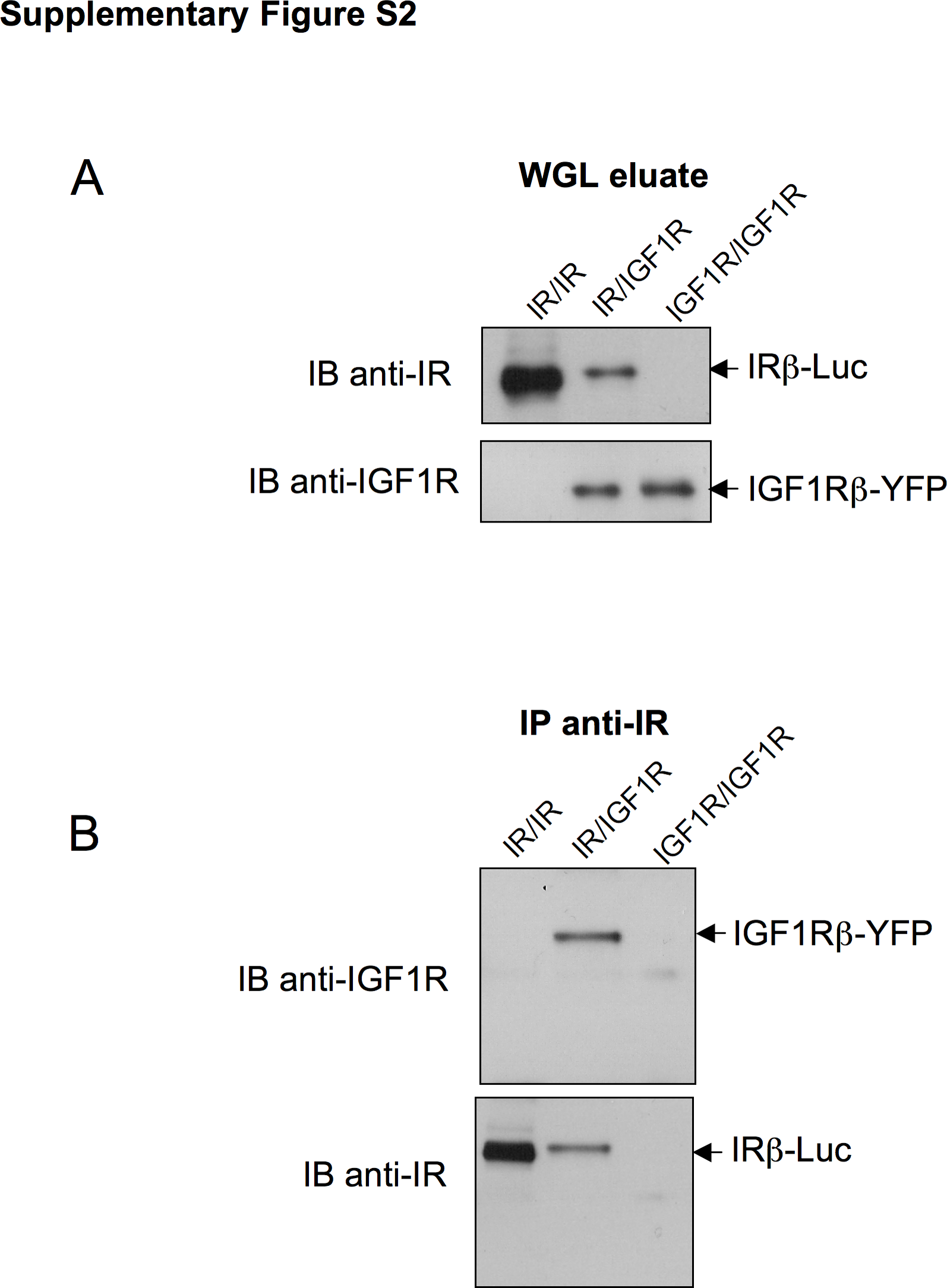

Supplement: Figure S2 — Validation of the method used for detection of IR/IGF1R hybrids. Partially purified receptors prepared from HEK-293 cells transfected as described in Fig. 1 were used to establish the specificity of the immunoprecipitation and immunoblotting experiments. (A) WGL eluates were submitted to SDS-PAGE followed by immunoblotting (IB) using anti-IR (Santa Cruz C19) or anti-IGF1R antibodies (Santa Cruz C-20). (B) Luciferase and YFP-tagged IR, IGF1R and hybrid receptors were immunoprecipitated (IP) using an anti-IR antibody (CT1) immobilized on sepharose beads, submitted to SDS-PAGE followed by immunoblotting using anti-IGF1R (Santa Cruz C-20) and anti-IR (Santa Cruz C-19) antibodies. After immunoprecipitation with anti-IR antibody, only hybrid receptors were detected when immunoblotting with anti-IGF1R antibody. (TIF) [file pone.0041992.s002.tif]
